# Differential brainstem connectivity according to sex and menopausal status in healthy men and women

**DOI:** 10.21203/rs.3.rs-4875269/v1

**Published:** 2024-08-12

**Authors:** Lisa A Kilpatrick, Arpana Gupta, David Meriwether, Swapna Mahurkar-Joshi, Vince W Li, Jessica Sohn, Juliana Reist, Jennifer S Labus, Tien Dong, Jonathan P Jacobs, Bruce D Naliboff, Lin Chang, Emeran A Mayer

## Abstract

Background Brainstem nuclei play a critical role in both ascending monoaminergic modulation of cortical function and arousal, and in descending bulbospinal pain modulation. Even though sex-related differences in the function of both systems have been reported in animal models, a complete understanding of sex differences, as well as menopausal effects, in brainstem connectivity in humans is lacking. This study evaluated resting-state connectivity of the dorsal raphe nucleus (DRN), right and left locus coeruleus complex (LCC), and periaqueductal gray (PAG) according to sex and menopausal status in healthy individuals. In addition, relationships between systemic estrogen levels and brainstem-network connectivity were examined in a subset of participants. Methods Resting-state fMRI was performed in 50 healthy men (age, 31.2 ± 8.0 years), 53 healthy premenopausal women (age, 24.7 ± 7.3 years; 22 in the follicular phase, 31 in the luteal phase), and 20 postmenopausal women (age, 54.6 ± 7.2 years). Permutation Analysis of Linear Models (5000 permutations) was used to evaluate differences in brainstem-network connectivity according to sex and menopausal status, controlling for age. In 10 men and 17 women (9 premenopausal; 8 postmenopausal), estrogen and estrogen metabolite levels in plasma and stool were determined by liquid chromatography-mass spectrometry/mass spectrometry. Relationships between estrogen levels and brainstem-network connectivity were evaluated by partial least squares analysis. Results Left LCC-executive control network (ECN) connectivity showed an overall sex difference (p = 0.02), with higher connectivity in women than in men; however, this was mainly due to differences between men and pre-menopausal women (p = 0.008). Additional sex differences were dependent on menopausal status: PAG-default mode network (DMN) connectivity was higher in postmenopausal women than in men (p = 0.04), and PAG-sensorimotor network (SMN) connectivity was higher in premenopausal women than in men (p = 0.03) and postmenopausal women (p = 0.007). Notably, higher free 2-hydroxyestrone levels in stool were associated with higher PAG-SMN and PAG-DMN connectivity in premenopausal women (p < 0.01). Conclusions Healthy women show higher brainstem-network connectivity involved in cognitive control, sensorimotor function, and self-relevant processes than men, dependent on their menopausal status. Further, 2-hydroxyestrone, implicated in pain, may modulate PAG connectivity in premenopausal women. These findings may relate to differential vulnerabilities to chronic stress-sensitive disorders at different life stages.

**Additional Declarations:** Competing interest reported. A.G. is a research consultant for YAMAHA. E.A.M. is a member of the scientific advisory boards of Danone, Axial Therapeutics, Amare, Mahana Therapeutics, Pendulum, Bloom Biosciences, and APC Microbiome Ireland. L.C. serves as an advisory board member or consultant for Ardelyx, Arena Pharmaceuticals, Bausch Health, Immunic, Ironwood Pharmaceuticals, Inc., Mauna Kea Technologies, and Trellus; and receives grant support from AnX Robotica, Arena Pharmaceuticals, and Ironwood Pharmaceuticals. All other authors declare no conflict of interest.

### PLAIN ENGLISH SUMMARY

Men and women show differences in the likelihood to develop chronic pain disorders, as well as anxiety and depression, which often accompany chronic pain, across the lifespan. Brainstem nuclei are tiny structures in the brain yet show powerful influences across the entire brain. Here, we investigated differences in the connectivity of several brainstem nuclei with major brain networks involved in stress responsiveness and pain modulation between healthy men and healthy premenopausal and postmenopausal women, as well as their association with estrogen levels in the body. We found higher connectivity between the locus coeruleus and the executive control network in women, especially premenopausal women, than in men, which may relate to differences in preferred information processing strategies and anxiety symptoms. We also found higher connectivity between the periaqueductal gray and the default mode network in postmenopausal women and the sensorimotor network in premenopausal women than in men. Further, higher levels of 2-hydroxyestrone, an estrogen metabolite that plays a role in visceral pain sensitivity, was related to periaqueductal gray connectivity in premenopausal women. These latter findings may relate to differences in vulnerability to visceral pain disorders at different life stages. These results help in identifying potential contributors to sex differences in vulnerability to chronic disorders that reduce the quality of life, before a disorder develops.

### HIGHLIGHTS

- We evaluated differences in the connectivity of several brainstem nuclei with major brain networks between healthy premenopausal and postmenopausal women and men, as well as relationships between connectivity and estrogen levels in plasma and stool.
- Premenopausal women showed higher connectivity between the left locus coeruleus complex and executive control network compared to that in men, and higher connectivity between the periaqueductal gray and sensorimotor network compared to that in men and postmenopausal women.
- Postmenopausal women showed higher connectivity between the periaqueductal gray and default mode network compared to that in men.
- Levels of free 2-hydroxyestrone, an estrogen implicated in visceral pain, in plasma and stool were associated with periaqueductal gray connectivity in premenopausal women.
- These results may relate to differential vulnerabilities to chronic pain and stress-sensitive disorders across the lifespan.

### BACKGROUND

Brainstem nuclei, including the locus coeruleus complex (LCC), dorsal raphe nucleus (DRN), and periaqueductal gray (PAG), play critical roles in ascending monoaminergic modulation of brain and vital functions, as well as in endogenous descending pain modulation. Alterations in these modulations have been demonstrated in chronic pain disorders, as well as in anxiety and depression, which are often comorbid with each other [1–5]. The LCC is the primary source of noradrenergic innervation of the forebrain and exerts a powerful modulatory role over cognitive and affective functions via widespread cortical and subcortical projections. [6] The DRN is the primary serotonergic nucleus in the central nervous system (CNS) and modulates several vital functions, including mood, appetite, and sleep, through ascending projections to many cortical and subcortical brain regions. [7]. The PAG is involved in integrated descending modulation of pain, as well as in autonomic and behavioral responses to threat. It has reciprocal connections with prefrontal and emotion-regulation regions and receives top-down input from the orbitofrontal cortex and insula [4].

Sex differences in LCC, DRN, and PAG structure and function exist [8, 9]. For instance, the LCC is more sensitive to stressors in women than men [10, 11]. In addition, men show stronger LCC modulation of the hippocampus than women [12], consistent with animal research demonstrating greater LCC noradrenergic input to the hippocampus in males than in females [13]. Animal research indicates PAG/DRN involvement in sex differences in pain-related behaviors, promoting anti-nociception in males and pain-related locomotor behaviors in females [9]. Women have greater risk for numerous chronic pain disorders, as well as greater risk for anxiety and depression [14]. Sex differences in the connectivity of brainstem nuclei may be related to differential vulnerability to chronic pain conditions and comorbid mood disorders.

Estrogens are known to impact LCC, DRN, and PAG function. Estrogens modulate LCC output, generally increasing noradrenergic levels in target regions [15]. Estrogens also increase the expression of tryptophan hydroxylase, a rate-limiting enzyme for serotonergic synthesis, in the DRN, reducing anxiety and increasing active coping behavior in animal studies [16–18]. The PAG contains a large population of estrogen receptors, contributing to known sex differences in response to morphine administration, with a greater antinociceptive effect in men than in women [19].

Menopause is associated with increased risk for anxiety and depression [20], as well as for some chronic pain conditions, such as fibromyalgia, migraine, and back pain [21]. However, the role of estrogen in the severity of symptoms in disorders of gut brain interaction (DGBI) in premenopausal and postmenopausal women is incompletely understood. We previously found that postmenopausal women with irritable bowel syndrome (IBS) have greater overall IBS symptom severity and worse health-related quality of life [22]. However, in a large global epidemiology study in individuals with IBS and other DGBI, premenopausal women reported a greater frequency of gastrointestinal symptoms compared to men and postmenopausal women [23]. In addition, another study found that menopause may be associated with less risk for DGBI such as IBS [24]. Changes in brainstem connectivity during menopause may contribute to these changes in symptom severity in postmenopausal women. However, a complete understanding of sex and menopausal effects on brainstem connectivity, especially in humans, is lacking.

In the current study, we aimed to evaluate sex and menopausal status effects on resting-state connectivity of the DRN, left and right LCC, and PAG with major brain networks involved in stress responsiveness, pain modulation, and emotion regulation, including the central autonomic network (CAN), default mode network (DMN), emotional arousal network (EAN), executive control network (ECN), salience network (SAL), and sensorimotor network (SMN), in healthy individuals. In addition, relationships between estrogen levels and brainstem-network connectivity were examined in a subset of participants.

## METHODS

### Participants

Healthy men and women were recruited from the Los Angeles area through advertisements and local clinics. Exclusion criteria were as follows: chronic pain disorder including IBS, major neurological condition or vascular disease, current or past psychiatric illness, substance use disorder, use of centrally acting medications, pregnant or breastfeeding, weight > 400 lbs, and metal implants. In addition, individuals with < 8 minutes of low-motion resting-state fMRI data (with low motion defined as framewise displacement < 0.2 mm) were excluded.

All participants underwent a medical history and physical examination. Participants also completed the Hospital Anxiety and Depression (HAD) scale to assess their acute mental state [25]. Menopausal status was determined by the following criteria: women who had regular menses within the previous 12 months were premenopausal; women who did not have menses within the previous 12 months, with decreased estradiol (E2) and increased follicle-stimulating hormone levels based on normal laboratory values were considered postmenopausal. Among premenopausal women, menstrual cycle phase was determined using urine ovulation kits. Pregnancy or childbirth within the past 12 months were exclusion criteria for all women.

This study was approved by the Institutional Review Board (IRB) at the University of California, Los Angeles's Office of Protection for Research Subjects (Nos. 20–000540 and 20–000515). All participants provided written informed consent.

### Imaging acquisition and preprocessing

Participants underwent neuroimaging on a 3.0 T Siemens Prisma MRI scanner (Siemens, Erlangen, Germany). Structural MRI (T1-weighted and T2-weighted) and resting-state fMRI scans were performed in accordance with Human Connectome Project (HCP) protocols (version 4.3). Specifically, acquisition parameters for high-resolution T1-weighted scans were as follows: echo time, 1.81 ms; repetition time, 2500 ms; slice thickness, 0.8 mm; number of slices, 208; voxel matrix, 320 × 300; and voxel size, 1.0 × 1.0

× 0.8 mm. Parameters for the T2-weighted scans were as follows: echo time, 564 ms; repetition time, 3200 ms; slice thickness, 0.8 mm; number of slices, 208; voxel matrix, 320 × 300; and voxel size, 1.0 × 1.0 × 0.8 mm. Resting-state scans were obtained in anterior-posterior and posterior-anterior directions (8 minutes each; total duration, 16 minutes), with eyes open. Parameters for the resting-state scan were as follows: echo time, 37 ms; repetition time, 1000 ms; flip angle, 52 deg; slice thickness, 2 mm; number of slices, 88; voxel matrix, 104 × 104; and voxel size, 2.0 × 2.0 × 2.0 mm. Spin echo fieldmaps were also acquired in anterior-posterior and posterior-anterior directions for distortion correction.

Images were preprocessed using the ABCD-HCP pipeline (https://github.com/DCAN-Labs/abcd-hcp-pipeline) [26], which is based on the HCP pipeline [27]. Briefly, structural images underwent bias field correction, volume segmentation and cortical surface reconstruction using FreeSurfer 6.0 [28], and MNI registration; resting-state images underwent distortion and bias field correction, realignment, MNI registration via T1-weighted registration, and intensity normalization. Additionally, resting-state images were denoised by regressing out head motion parameters and white matter and cerebrospinal fluid signals, which were refined using respiratory motion filtering (13.2–18.6 breaths per minute), and band-pass filtering (0.008, 0.09 Hz) [29].

Denoised resting-state images were parcellated using the Destrieux atlas for cortical regions [30], Harvard-Oxford atlas for subcortical regions, and ascending arousal network (AAN) atlas for brainstem regions [31]. Fisher-transformed connectivity matrices were created using 8 min of low-movement data (defined as framewise displacement $< .20$ mm). The correlation between each brainstem region of interest (DRN, LCL, LCR, and PAG) and each network of interest (central autonomic network [CAN], default mode network [DMN], emotional arousal network [EAN], executive control network [ECN], salience network [SAL], and sensorimotor network [SMN]) was calculated as the average of the pairwise correlations between the brainstem region and all of regions belonging to the network. Regions included in each network of interest are indicated in Table 1.

Table 1
Network definitions based on the Destrieux cortical and Harvard-Oxford subcortical atlases

| Network | Brain regions | Atlas labels |
|---|---|---|
| Central Autonomic Network (CAN) | Orbitofrontal cortex, anterior cingulate cortex, anterior insula, amygdala, midbrain nuclei | transverse frontopolar gyri and sulci, middle-anterior part of the cingulate gyrus and sulcus,<br><br>short insular gyri, orbital gyri, gyrus rectus, subcallosal gyrus, anterior segment of the circular sulcus of the insula, lateral orbital sulcus, medial orbital sulcus, orbital sulci, amygdala, ventral diencephalon |
| Default Mode Network (DMN) | Medial prefrontal cortex, posterior cingulate cortex, inferior parietal cortex, lateral temporal cortex | transverse frontopolar gyri and sulci, posterior-dorsal part of the cingulate gyrus, posterior-ventral part of the cingulate gyrus,<br><br>angular gyrus, supramarginal gyrus, precuneus, lateral aspect of the superior temporal gyrus, planum temporale |
| Emotional Arousal Network (EAN) | Medial and ventrolateral prefrontal cortex, anterior cingulate cortex, parahippocampal gyrus, hippocampus, amygdala | transverse frontopolar gyri and sulci, anterior part of the cingulate gyrus and sulcus, opercular part of the inferior frontal gyrus, orbital part of the inferior frontal gyrus, triangular part of the inferior frontal gyrus, parahippocampal gyrus, gyrus rectus, subcallosal gyrus, suborbital sulcus, hippocampus, amygdala |
| Executive Control Network (ECN) | Dorsolateral prefrontal cortex, superior parietal cortex | middle frontal gyrus, superior parietal lobule, inferior frontal sulcus, middle frontal sulcus, intraparietal sulcus and transverse parietal sulci, subparietal sulcus |
| Salience Network (SAL) | Orbitofrontal cortex, anterior mid-cingulate cortex, anterior insula | transverse frontopolar gyri and sulci, middle-anterior part of the cingulate gyrus and sulcus, short insular gyri, orbital gyri, gyrus rectus, anterior segment of the circular sulcus of the insula, lateral orbital sulcus, medial orbital sulcus, orbital sulci |
| Sensorimotor Network (SMN) | Primary/secondary somatosensory cortices, primary/supplementary motor cortices, posterior mid-cingulate cortex, posterior insula, thalamus, basal ganglia | paracentral lobule and sulcus, subcentral gyrus and sulci, middle-posterior part of the cingulate gyrus and sulcus, superior frontal gyrus, long insular gyrus and central sulcus of the insula, postcentral gyrus, precentral gyrus, central sulcus, inferior segment of the circular sulcus of the insula, superior segment of the circular sulcus of the insula, superior frontal sulcus, postcentral sulcus, inferior part of the precentral sulcus, superior part of the precentral sulcus, thalamus, caudate, putamen, pallidum, nucleus accumbens |

## Determination of free and total estrogens in plasma and stool

In a subset of participants (10 men; 9 premenopausal women, with 4 and 5 in follicular and luteal phases, respectively, during scanning and sample collection; and 8 postmenopausal women), the levels of free and total estrogens and estrogen metabolites in plasma and stool were determined by liquid chromatography-mass spectrometry/mass spectrometry (LC-MS/MS). Estrogen species analyzed included estrone (E1), estradiol (E2), estriol (E3) as well as 12 additional estrogen metabolites: 2-hydroxyestrone (2OHE1), 2-methoxyestrone (2MeOE1), 2-hydroxyestradiol (2OHE2), 2-methoxyestradiol (2MeOE2), 2-hydroxyestrone-3-methyl ether (3MeOE1), 4-hydroxyestrone (4OHE1), 4-methoxyestrone (4MeOE1), 4-methoxyestradiol (4MeOE2), 16α-hydroxyestrone (16aOHE1), 17-epiestriol (17epiE3), 16-ketoestradiol (16ketoE2), and 16-epiestriol (16epiE3). Both sample preparation and LC-MS/MS methods followed previously published methods [32–34], with minor modifications.

Plasma sample preparation. Plasma was prepared with and without enzymatic hydrolysis. For the determination of free estrogen, 100 uL plasma was combined with stable heavy isotope internal standards and 300 uL of basic reaction buffer (BRB) consisting of 0.15 M acetate buffer pH 4.5 containing 1.0 mg/mL L-ascorbic acid. For the determination of total estrogen, 100 uL plasma was prepared as above, but 15 uL of β-glucuronidase/aryl-sulfatase (from Helix pomatia) was also added and samples were kept gently rocking at 37°C overnight. For both free and total samples, isopropanol was added (5% v/v) and lipids were extracted with 400 cc supported liquid extraction (SLE) cartridges (Biotage LLC) using 3 x 1.5 mL dichloromethane extractions and dried under argon.

Stool sample preparation. Stool for both free and total estrogen (100 mg each) was combined with internal standards and 1 mL BRB and subjected to bead mill homogenization (Biotage Lysera). Stool for total estrogen was combined with 15 uL deconjugation enzyme and reacted overnight as above. Both sample sets were combined with 500 ul acetonitrile; re-homogenized; and centrifuged. Supernatant was loaded into 2 cc SLE cartridges (Biotage) and extracted with 3 x 2 mL DCM followed by drying under argon.

Sample derivatization. Dried plasma and stool samples were combined with 100 uL of 1 mg/mL-acetone dansyl chloride and 100 uL 0.1 M sodium bicarbonate buffer pH 9.2; reacted for 20 min at 60°C; centrifuged; and transferred to LCMS vials.

LC-MS/MS. LC-MS/MS analysis was performed on an Agilent 1290/SCIEX QTrap 5500 system using principles of tuning, method validation, quality control, calibration, and quantitation that we previously described [35]. MS/MS settings for derivatized estrogens and stable heavy isotope internal standards were determined using reference calibration and internal standards (Steraloids); values were comparable to those previously reported [32, 33]. Liquid chromatography was performed using a Kinetex C18 1.7 um 2.1 mm x 150 mm column (Phenomenex), while the gradient transitioned from 90% water/0.1% formic acid to 95% acetonitrile/0.1% formic acid across 20 minutes. Concentration of all estrogens was determined as ng/mL-plasma or ng/5 mg-stool.

### Statistical analysis

Permutation Analysis of Linear Models (PALM) with non-parametric combination (NPC) was used to evaluate brainstem-network connectivity according to sex and menopausal status, with 5000 permutations [36, 37]. NPC combines the test statistics of separate analyses into a single joint statistic, the significance of which is assessed through synchronized permutations for each of the separate tests [37, 38]. In this case, the test statistics of separate analyses of men vs women in follicular phase, men vs. women in the luteal phase and men vs postmenopausal women, controlling for age, were combined into a joint statistic representing men vs women across menstrual status/phase categories (i.e., an overall sex difference) and joint statistics representing differences according to menopausal status (i.e., premenopausal women vs men, premenopausal women vs postmenopausal women, and postmenopausal women vs men). Familywise error (FWE)-corrected p-values $< 0.05$ were considered significant.

Partial least squares correlation (PLSC) analysis was applied to examine relationships between estrogen levels and brainstem-network connectivity with significant sex/menopausal status effects, using plscmd in Matlab (http://www.rotman-baycrest.on.ca/pls) [39]. PLSC analysis is a multivariate analytical technique that identifies weighted patterns of variables in two blocks of variables that maximally covary with each other and is appropriate for data with multicollinearity. As metabolite pathways are interrelated, multicollinearity in the estrogen data was expected. In the present study, Block 1 comprised brainstem-network data and Block 2 comprised estrogen data (free and total levels in plasma and stool). Bootstrap estimation was applied (5000 samples), and bootstrap ratios of magnitude 2.58 or greater (corresponding to $p < 0.01$) were considered significant.

## RESULTS

### Participant characteristics

From the 50 men and 75 women (21 postmenopausal), 3 men and 2 women (1 postmenopausal) were excluded from analysis due to insufficient low-motion resting-state data. Accordingly, 50 healthy men (mean age: 31.2 ± 8.0 years), 53 healthy premenopausal women (mean age: 24.7 ± 7.3 years), and 20 healthy postmenopausal women (mean age: 54.6 ± 7.2 years) were included in the analysis. Among the premenopausal women, 22 were scanned during the follicular phase and 31 were scanned during the luteal phase of their menstrual cycle. None of the postmenopausal women were taking hormone replacement therapy at the time of scanning. Although HAD anxiety and depression scores were low in this healthy population, analysis of variance revealed significant differences in HAD anxiety ($F(2,115) = 4.09$, $p = 0.02$), with significantly higher scores in premenopausal women than in men ($p = 0.005$) (Table 2).

Table 2
Participant characteristics

| | N | Age (yr) | HAD Anxiety | HAD Depression |
|---|---|---|---|---|
| **Men** | 47 | 31.2 ± 8.0 | 3.0 ± 2.1 | 1.4 ± 1.6 |
| **Premenopausal women** | 51 | 24.7 ± 7.3 | 4.5 ± 2.9 | 1.3 ± 1.6 |
| **Postmenopausal women** | 20 | 54.6 ± 7.2 | 3.4 ± 2.9 | 0.9 ± 1.1 |
| **Data are shown as mean ± standard deviation; HAD, Hospital Anxiety and Depression** | | | | |

## Sex and menopausal status effects on brainstem connectivity

Significant differences in brainstem-network connectivity according to sex and menopausal status are summarized in Fig. 1.

NPC analysis revealed a significant overall sex difference in left LCC-ECN connectivity ($p_{fwe}$=0.02), with higher connectivity in all women than in men. However, subgroup analysis according to menopausal status revealed that the overall sex difference was mainly due to significantly higher connectivity in premenopausal women than in men ($p_{fwe}$=0.008). No significant difference was observed between postmenopausal women and men ($p_{fwe}$=0.69). There was a trend toward higher connectivity in premenopausal women than in postmenopausal women ($p_{fwe}$=.051).

NPC analysis revealed significant sex differences in PAG connectivity dependent on menopausal status. Specifically, PAG-DMN connectivity was significantly higher in postmenopausal women compared to me, a difference not seen between premenopausal women and men ($p_{fwe}$=0.03; $p_{fwe}$=0.35, respectively). Additionally, PAG-SMN connectivity was significantly higher in premenopausal women than in men ($p_{fwe}$=0.03) and postmenopausal women ($p_{fwe}$=0.007).

## Relationships between estrogens and brainstem connectivity

As indicated above, left LCC-ECN, PAG-DMN, and PAG-SMN connectivity showed significant group differences and were, thus, submitted to PLSC analysis in a subset of participants with estrogen/estrogen metabolite data to evaluate relationships between connectivity and estrogen levels within each group. Specifically, the three connections, along with estrogen/estrogen metabolite levels in plasma and stool, were simultaneously submitted to PLSC analysis in each participant group (i.e. men, premenopausal women, postmenopausal women). A summary of estrogen levels is provided in Fig. 2; as expected, levels were lower, with a more restricted range, in men and postmenopausal women compared to that in premenopausal women.

PLSC analysis of connectivity-estrogen relationships in men revealed that higher levels of free 2MeOE2 in plasma, and free and total 3MeOE1 in stool, were significantly associated with higher left LCC-ECN and PAG-DMN connectivity (first latent variable, accounting for 47.3% of the cross-block variance) (Fig. 3). No other latent variables reached significance.

PLSC analysis of connectivity-estrogen relationships in premenopausal women revealed that higher levels of free E1 and 2OHE1 in plasma and free 2OHE1 and 4OHE1 in stool, were significantly associated with higher PAG-DMN and PAG-SMN connectivity (first latent variable, accounting for 43.9% of the cross-block variance), while lower levels of total 2OHE2 and 4MeOE1 in plasma, and lower levels of free 4MeOE2 in stool, were associated with higher left LCC-ECN connectivity (second latent variable, accounting for 36.8% of the cross-block variance) (Fig. 4).

PLSC analysis of connectivity-estrogen relationships in postmenopausal women revealed that higher levels of total E2 and lower levels of free 16aOHE1 in plasma, and higher levels of free 16epiE3, total 3MeOE1, and free and total overall levels of estrogen (summation of all estrogens and estrogen metabolites) in stool, were significantly associated with higher left LCC-ECN connectivity (first latent variable, accounting for 43.8% of the cross-block variance), while higher levels of free E2, total 16aOHE1, and free and total overall levels of estrogen in plasma were associated with higher PAG-SMN connectivity (second latent variable, accounting for 30.2% of the cross-block variance) (Fig. 5).

## DISCUSSION

The present study evaluated differences in resting-state connectivity between specific brainstem nuclei (DRN, left and right LCC, and PAG) and key brain networks (CAN, DMN, EAN, ECN, SAL, and SMN) implicated in stress responsiveness and pain modulation according to sex and menopausal status in healthy individuals. We found a significant overall sex difference in left LCC-ECN connectivity, with generally higher connectivity in women than in men, mainly driven by higher connectivity in premenopausal women. There was also significantly higher PAG-SMN connectivity in premenopausal women than in men and in premenopausal women than postmenopausal women. In contrast, significantly higher PAG-DMN connectivity was present in postmenopausal women than in men. Further, relationships between left LCC-ECN, PAG-SMN, and PAG-DMN and estrogen levels in plasma and stool were observed.

### Sex differences in LCC resting-state connectivity may relate to differences in information processing priorities

The left LCC showed higher connectivity with the ECN in women, especially premenopausal women, than in men. The ECN comprises lateral prefrontal and parietal regions and supports executive functions such as working memory, selective attention, and cognitive control [40]. Animal and human studies suggest that enhanced functional coupling of the LCC with the ECN is associated with increased goal-directed attention and decreased impulsivity [41, 42].

LCC noradrenergic signaling biases perception, attention, and memory toward more salient stimuli by selectively amplifying the activity of priority mechanisms operating at the moment [43]. Thus, the present results may relate to basic sex-related biases in information processing priorities.

Substantial evidence suggests that while men show a greater preference for allocentric knowledge (e.g., describing objects/others independent of one's own perspective), with a greater reliance on hippocampal-based strategies [8], women show a greater preference for egocentric knowledge (e.g., describing objects in terms of one's own spatial perspective and using more privileged information in inference), with a greater reliance on working memory processes mediated by frontal regions, such as those in the ECN [8, 44]. In the absence of any specific task or stimuli (i.e., resting-state conditions), this greater focus on egocentric knowledge may underlie the observed difference in left LCC connectivity between men and women. In addition, anxiety and egocentricism are related, with greater reliance on egocentric perspective-taking/mentalizing in those experiencing anxiety [45]. Thus, greater LCC-ECN in premenopausal women than in men may offer benefits in terms of increased goal-directed attention but may hinder mentalizing under stress, increasing self-focus. This may be related to the higher current anxiety symptom scores in premenopausal women than in men in the present study.

Interestingly, unlike the left LCC, the right LCC did not show any sex differences in connectivity. A recent mixed-sex study of LCC connectivity gradients found greater relationships between age, anxiety/depression symptoms, and cognitive performance for the left LCC than for the right LCC [46]. Additional research suggests that neurodegenerative disorders affect the left LCC more than the right LCC [47, 48]. Thus, the left LCC may be more pliable or sensitive than the right LCC. However, additional research is needed.

In premenopausal women, decreased levels of mainly metabolites in the 2-hydroxylation pathway of estrogen metabolites, including plasma 2OHE2, were associated with higher left LCC-ECN connectivity. Metabolites of the 2-hydroxylation pathway are generally considered to have weak estrogenic activity; however, 2OHE2 has structural similarities to catecholamines and can compete with noradrenaline in the brain [49]. Thus, in premenopausal women, circulating 2OHE2 may interact with LC noradrenergic output, modulating left LCC-ECN connectivity. In contrast, in men, higher overall levels of methylated estrogen metabolites, including 2MeOE2 in plasma, and in postmenopausal women, higher levels of total E2 in plasma, were associated with higher left LCC-ECN connectivity (i.e., more 'feminine' connectivity). 2MeOE2 was one of the more abundant estrogen metabolites in plasma in men in the present study and E2 is a major endogenous estrogen. These results suggest that estrogens may affect LCC-ECN connectivity in men and postmenopausal women, but the effects may be more apparent for metabolites with relatively higher levels, as levels were generally lower in these individuals.

### Sex differences in PAG resting-state connectivity may relate to sex differences in pain processing

The PAG showed higher connectivity with the DMN in postmenopausal women than in men. The DMN comprises the medial prefrontal cortex, posterior cingulate cortex, precuneus, inferior parietal cortices, and lateral temporal cortices, and is involved in self-referential processes [50, 51]. Given the role of the PAG in threat-related processing, this result may reflect an enhanced link between self- and threat-related processing in postmenopausal women.

The PAG also showed higher connectivity with the SMN in premenopausal women than in men and postmenopausal women. The present results are consistent with a previous neuroimaging study that reported greater PAG connectivity with sensorimotor-related brain regions in healthy women than in healthy men [52], and add to the literature by showing a dependence on menopausal status.

The SMN comprises sensorimotor, mid-cingulate and superior frontal cortices, as well as the posterior insula, thalamus, and basal ganglia [53, 54]. The SMN is involved in central processing and modulation of visceral and somatic sensory information and both the PAG and SMN are involved in pain processing. Previous studies in patient populations indicate that increased connectivity of the PAG with the SMN, or specific regions within the SMN, may be associated with an increased risk of the development of chronic pain following mild traumatic brain injury [55] and increased central sensitization symptoms in patients with fibromyalgia [56]. Our findings also align with an increased vulnerability to chronic pain conditions such as IBS in women, as well as a higher frequency of gastrointestinal symptoms in premenopausal women than in men and postmenopausal women [23, 57]. However, a mixed-sex study in healthy individuals reported that resting-state connectivity between the PAG and SMN positively correlated with conditioned pain modulation, suggesting more efficient endogenous pain modulation with increased connectivity [58]. Primary somatosensory cortex out modulates sensory gain and nociception, with layer 5 outputs to subcortical targets, including the PAG, comprising an anti-nociceptive pathway, and layer 6 outputs to the thalamus, which is also a component of the SMN and interacts with the PAG, comprising a pro-nociceptive pathway [59]. Thus, the interpretation of increased PAG-SMN connectivity is complicated and may require a finer-grained analysis.

However, one notable finding in the analysis of connectivity-estrogen relationships was that, in premenopausal women, increased plasma and stool free 2OHE1 was associated with increased PAG-SMN and PAG-DMN connectivity. 2OHE1 has been shown to increase nociceptor activation via transient receptor potential ankyrin 1 (TRPA1) and transient receptor potential vanilloid type-1 (TRPV1) channels in a mouse model of uterine pain [60]. Peripheral TRPA1 and TRPV1 sensitization have been implicated in visceral pain disorders, including DGBI [61–63]. However, central factors may be necessary for the persistence of visceral hypersensitivity [64]. TRPV1 receptors are expressed in the PAG and antagonists applied to the dorsolateral PAG can reduce anxiety-like and nociceptive behavior in animal models [65, 66]. Thus, estrogen metabolites may modulate the increased PAG-SMN and PAG-DMN connectivity in premenopausal women predisposing them to chronic pain disorders, such as DGBI.

## Limitations

The present study has several limitations. The number of postmenopausal women was relatively small, limiting the power to detect differences between postmenopausal and premenopausal women and sex differences that emerge or reverse after menopause. Additionally, we investigated the overall connectivity of the LCC, DRN, and PAG, without consideration of differential connectivity within each of these brainstem regions. Although small, these regions show variations in connectivity, with a rostral-caudal connectivity gradient in the LC and subregions in the DRN and PAG with differential connectivity supporting various functions [67]. This may have contributed to the lack of significant sex differences in DRN connectivity. However, a finer-grained analysis is beyond the scope of the present study. Additionally, estrogen/estrogen metabolite data were available in a limited subset of participants; thus, further research is required to confirm the present findings. Finally, as a major limitation of correlational studies, the causality or directionality of interactions could not be addressed.

## Conclusions

The present study expands the limited research on sex and menopausal effects on brainstem connectivity, and their relationships with various estrogens, in humans. We found that healthy women show higher left LCC and PAG connectivity with networks involved in cognitive control, and sensorimotor function and self-relevant processes, respectively, than men, dependent on their menopausal status. Although such differences may show benefits under optimal conditions, they may also relate to differential vulnerabilities to chronic pain and stress-sensitive disorders at different life stages. In particular, PAG connectivity with the SMN and DMN may be modulated by circulating 2OHE1 in premenopausal women, potentially via TRPV1 signaling, contributing to peripheral and central sensitization processes and increasing the risk for chronic pain disorders, such as DGBI.

## Declarations

**Ethics approval and consent to participate:** This study was approved by the Institutional Review Board (IRB) at the University of California, Los Angeles's Office of Protection for Research Subjects (Nos. 20-000540 and 20-000515). All participants provided written informed consent.

**Consent for publication:** Not applicable

**Availability of data and materials:** Data are available from the corresponding authors on reasonable request.

**Competing interests:** A.G. is a research consultant for YAMAHA. E.A.M. is a member of the scientific advisory boards of Danone, Axial Therapeutics, Amare, Mahana Therapeutics, Pendulum, Bloom Biosciences, and APC Microbiome Ireland. L.C. serves as an advisory board member or consultant for Ardelyx, Arena Pharmaceuticals, Bausch Health, Immunic, Ironwood Pharmaceuticals, Inc., Mauna Kea Technologies, and Trellus; and receives grant support from AnX Robotica, Arena Pharmaceuticals, and Ironwood Pharmaceuticals. All other authors declare no conflict of interest.

**Funding:** NIH U54 DK123755 (EAM/LC)

**Authors' contributions:** L.A.K. performed the statistical analyses, prepared the figures, and drafted the manuscript; A.G. contributed to the study design and writing of the manuscript; D.M. provided the estrogen/metabolite data and contributed to the writing of the manuscript; S.M-J. contributed to the study design and writing of the manuscript, V.W.L. contributed to estrogen data analysis and the writing of the manuscript, J.S. contributed to data collection and writing of the manuscript, J.R. contributed to data collection and writing of the manuscript, J.S.L. contributed to the study design and writing of the manuscript, T.D. contributed to the study design and writing of the manuscript, J.P.J. contributed to the study design and writing of the manuscript, B.D.N. contributed to the study design and writing of the manuscript, L.C. procured funding and was a major contributor in the study design and writing of the manuscript, E.A.M. procured funding and was a major contributor in the study design and writing of the manuscript. All authors reviewed the manuscript.

**Acknowledgements:** Not applicable

## Figures

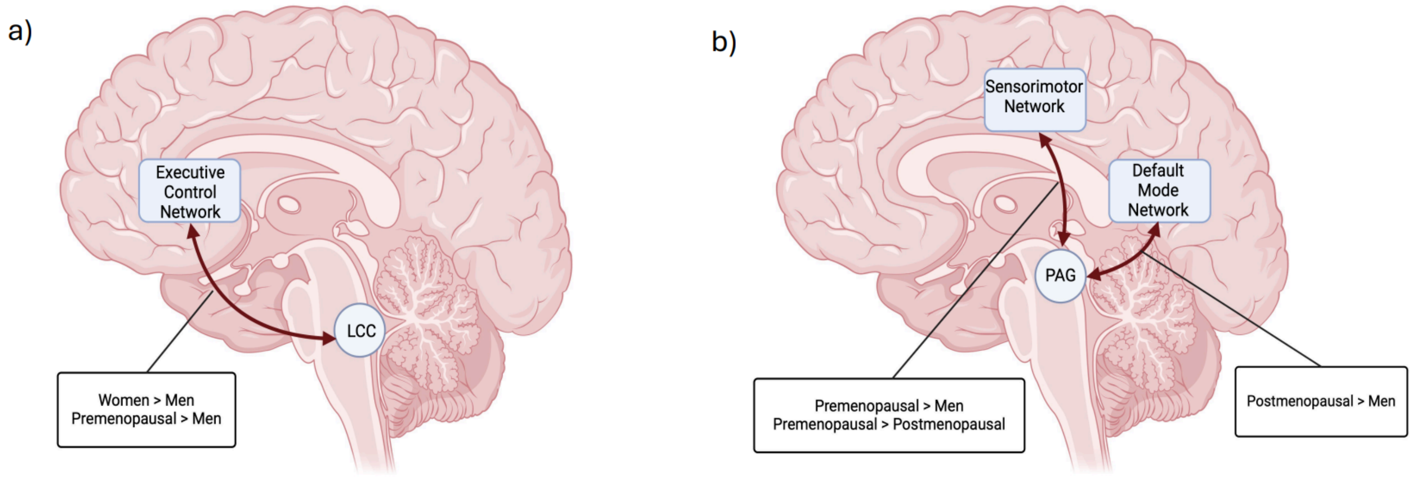

#### Figure 1

**Sex and menopausal status effects on brainstem connectivity.**(a) Brainstem-network connectivity showing a significant (familywise error-corrected) overall sex difference (all women vs men). Left LCC connectivity with the executive control network was higher in women than in men; however, a subgroup analysis indicated that this difference was mainly driven by premenopausal women. (b) Brainstem-network connectivity showing a significant (familywise error-corrected) difference between premenopausal and postmenopausal women and/or a sex difference dependent on menopausal status. PAG connectivity with the sensorimotor network was higher in premenopausal women than in men and postmenopausal women. In contrast, PAG connectivity with the default mode network was significantly higher in postmenopausal women than in men. LCC, locus coeruleus complex; PAG, periaqueductal gray

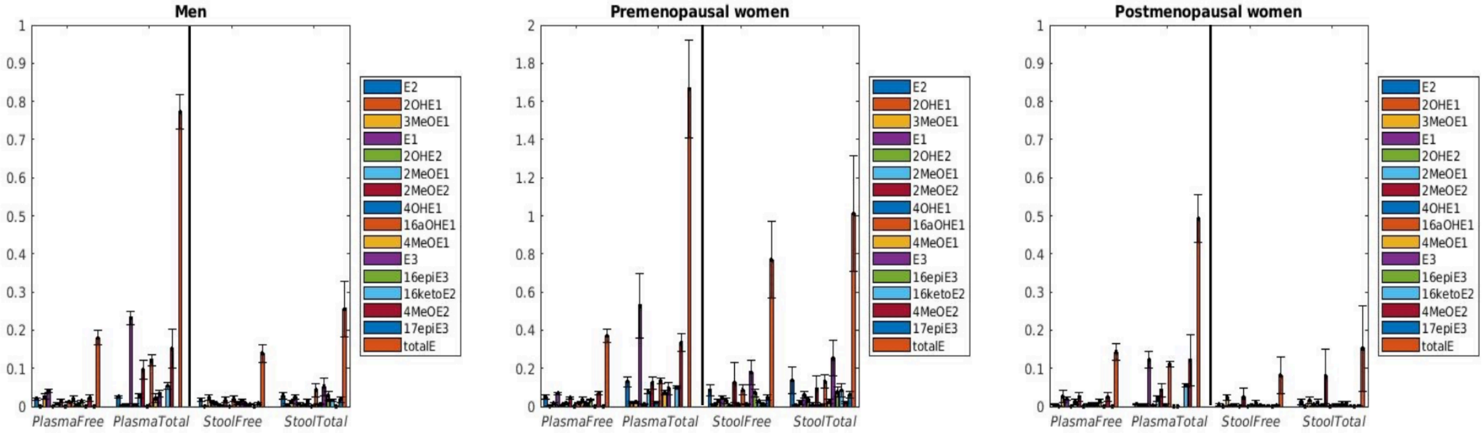

### Figure 2

**Mean estrogen levels, with standard deviations.** Plasma data is provided in units of ng/mL-plasma, while stool data is provided in units of ng/5mL-stool. E1, estrone; E2, estradiol; E3, estriol: 2OHE1, 2-hydroxyestrone; 2MeOE1, 2-methoxyestrone; 2OHE2, 2-hydroxyestradiol; 2MeOE2, 2-methoxyestradiol; 3MeOE1, 2-hydroxyestrone-3-methyl ether; 4OHE1, 4-hydroxyestrone; 4MeOE1, 4-methoxyestrone; 4MeOE2, 4-methoxyestradiol; 16aOHE1, 16α-hydroxyestrone; 17epiE3, 17-epiestriol; 16ketoE2, 16-ketoestradiol; 16epiE3, 16-epiestriol; totalE, total of all estrogens/metabolites

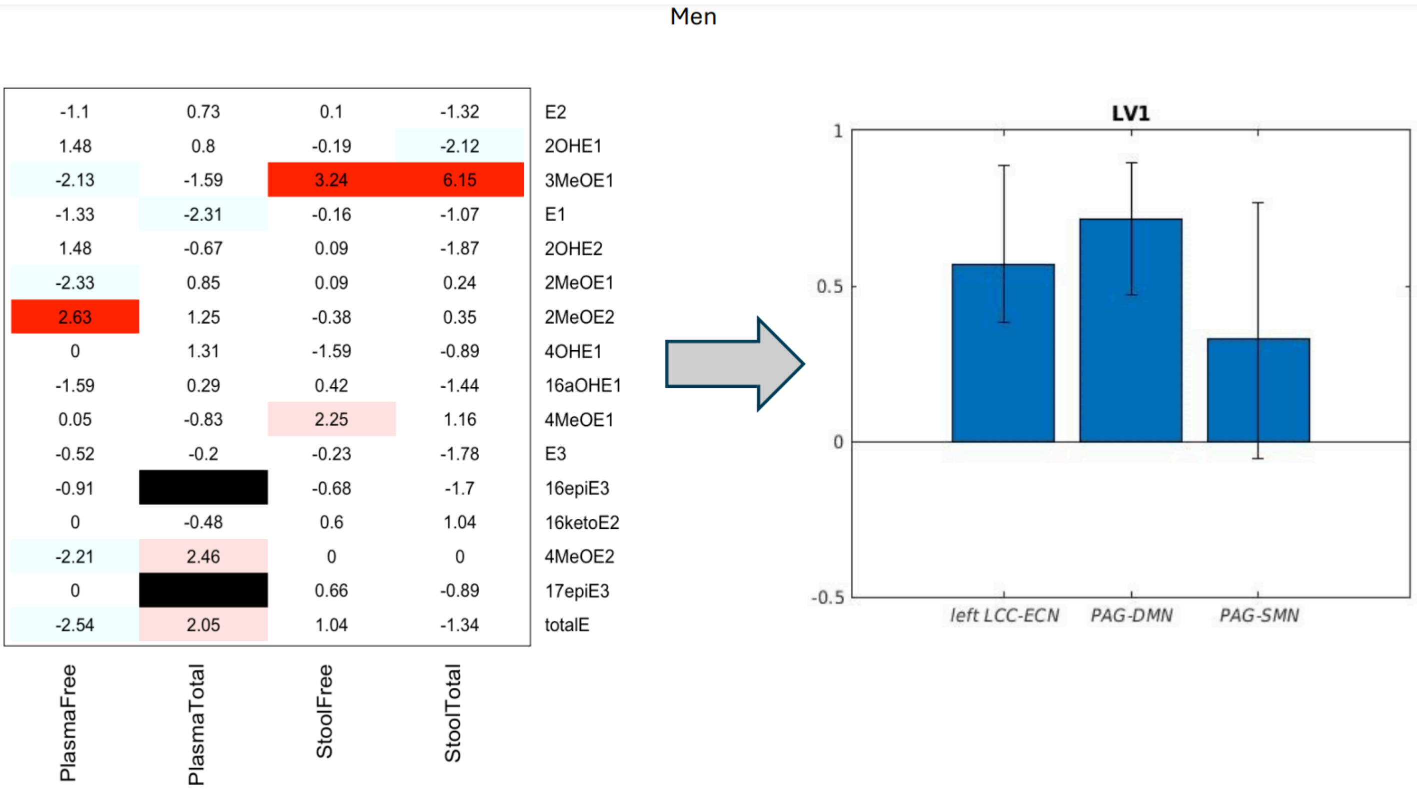

### Figure 3

**Relationships between estrogen/estrogen metabolite levels and connectivity in men.** In men, the first latent variable reflected estrogen levels associated with left LCC-ECN and PAG-DMN connectivity; no other associations reached significance. Heatmaps indicate the BSR of each evaluated estrogen/metabolite for the connectivity pattern shown in the corresponding bar graph, with significant ratios (at $p<0.01$) highlighted in dark blue (BSR<-2.58) or red (BSR>2.58). Additional estrogens/metabolites with a BSR meeting a lower significance threshold of $p<0.05$ are shown in light blue (BSR<-1.96) or pink (BSR>1.96). The plasma total levels of 16epiE3 and 17epiE3 were removed from analysis due to false positives (black bars). BSR, bootstrap ratio; DMN, default mode network; ECN, executive control network; LCC, locus coeruleus complex; PAG, periaqueductal gray; SMN, sensorimotor network; E1, estrone; E2, estradiol; E3, estriol: 2OHE1, 2-hydroxyestrone; 2MeOE1, 2-methoxyestrone; 2OHE2, 2-hydroxyestradiol; 2MeOE2, 2-methoxyestradiol; 3MeOE1, 2-hydroxyestrone-3-methyl ether; 4OHE1, 4-hydroxyestrone; 4MeOE1, 4-methoxyestrone; 4MeOE2, 4-methoxyestradiol; 16aOHE1, 16α-hydroxyestrone; 17epiE3, 17-epiestriol; 16ketoE2, 16-ketoestradiol; 16epiE3, 16-epiestriol; totalE, total of all estrogens/metabolites

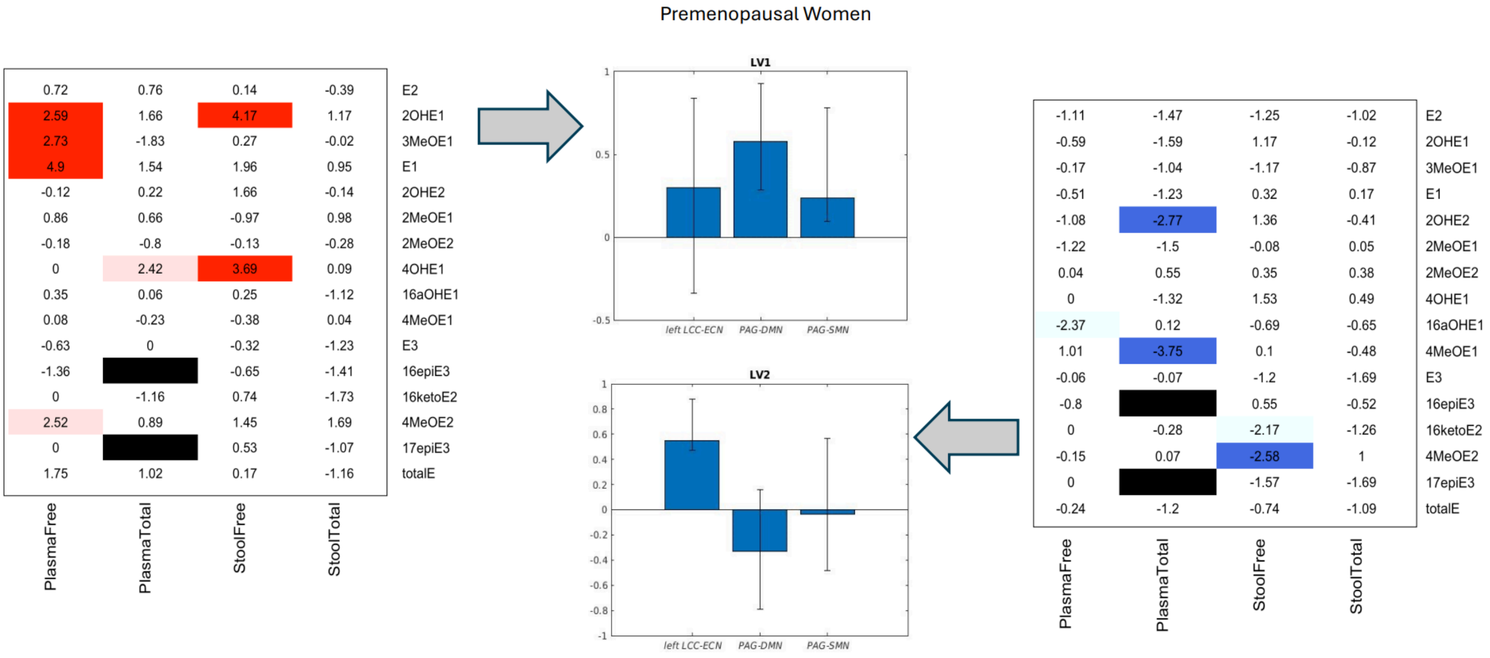

### Figure 4

**Relationships between estrogen/estrogen metabolite levels and connectivity in premenopausal women.** In premenopausal women, the first latent variable reflected estrogen levels significantly associated with PAG-SMN and PAG-DMN connectivity, while the second variable reflected estrogen levels significantly associated with left LCC-ECN connectivity. Heatmaps indicate the BSR of each evaluated estrogen/metabolite for the connectivity pattern shown in the corresponding bar graph, with significant ratios (at $p<0.01$) highlighted in dark blue (BSR<-2.58) or red (BSR>2.58). Additional estrogens/metabolites with a BSR meeting a lower significance threshold of $p<0.05$ are shown in light blue (BSR<-1.96) or pink (BSR>1.96). The plasma total levels of 16epiE3 and 17epiE3 were removed from analysis due to false positives (black bars). BSR, bootstrap ratio; DMN, default mode network; ECN, executive control; E1, estrone; E2, estradiol; E3, estriol: 2OHE1, 2-hydroxyestrone; 2MeOE1, 2-methoxyestrone; 2OHE2, 2-hydroxyestradiol; 2MeOE2, 2-methoxyestradiol; 3MeOE1, 2-hydroxyestrone-3-methyl ether; 4OHE1, 4-hydroxyestrone; 4MeOE1, 4-methoxyestrone; 4MeOE2, 4-methoxyestradiol; 16aOHE1, 16α-hydroxyestrone; 17epiE3, 17-epiestriol; 16ketoE2, 16-ketoestradiol; 16epiE3, 16-epiestriol; totalE, total of all estrogens/metabolites network; LCC, locus coeruleus complex; PAG, periaqueductal gray; SMN, sensorimotor network

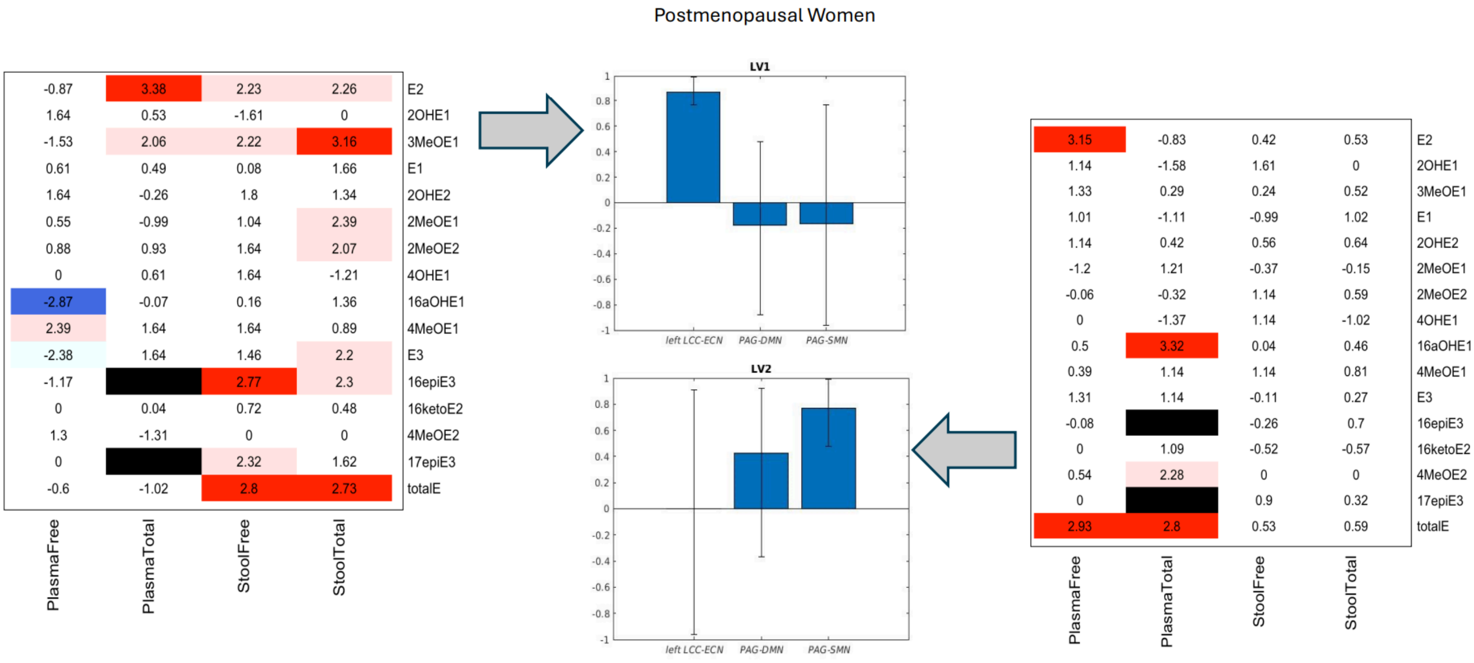

### Figure 5

**Relationships between estrogen/estrogen metabolite levels and connectivity in postmenopausal women.** In postmenopausal women, the first latent variable reflected estrogen levels significantly associated with left LCC-ECN connectivity, while the second variable reflected estrogen levels significantly associated with PAG-SMN connectivity. Heatmaps indicate the BSR of each evaluated estrogen/metabolite for the connectivity pattern shown in the corresponding bar graph, with significant ratios (at $p<0.01$) highlighted in dark blue (BSR<-2.58) or red (BSR>2.58). Additional estrogens/metabolites with a BSR meeting a lower significance threshold of $p<0.05$ are shown in light blue (BSR<-1.96) or pink (BSR>1.96). The plasma total levels of 16epiE3 and 17epiE3 were removed from analysis due to false positives (black bars). BSR, bootstrap ratio; DMN, default mode network; ECN, executive control network; LCC, locus coeruleus complex; PAG, periaqueductal gray; SMN, sensorimotor network; E1, estrone; E2, estradiol; E3, estriol: 2OHE1, 2-hydroxyestrone; 2MeOE1, 2-methoxyestrone; 2OHE2, 2-hydroxyestradiol; 2MeOE2, 2-methoxyestradiol; 3MeOE1, 2-hydroxyestrone-3-methyl ether; 4OHE1, 4-hydroxyestrone; 4MeOE1, 4-methoxyestrone; 4MeOE2, 4-methoxyestradiol; 16aOHE1, 16α-hydroxyestrone; 17epiE3, 17-epiestriol; 16ketoE2, 16-ketoestradiol; 16epiE3, 16-epiestriol; totalE, total of all estrogens/metabolites
